# Task Sharing in Global Cardiac Surgery: A Scoping Review

**DOI:** 10.1016/j.atssr.2022.12.004

**Published:** 2022-12-12

**Authors:** Laura DiChiacchio, Alejandra Castro-Varela, Dominique Vervoort, Amy G. Fiedler

**Affiliations:** 1 Division of Cardiothoracic Surgery, Department of Surgery, University of Utah, Salt Lake City, Utah; 2 Department of Cardiovascular Surgery, Mayo Clinic, Rochester, Minnesota; 3 Division of Cardiac Surgery, University of Toronto, Toronto, Ontario, Canada; 4 Institute of Health Policy, Management and Evaluation, University of Toronto, Toronto, Ontario, Canada; 5 Division of Cardiothoracic Surgery, Department of Surgery, University of California, San Francisco, San Francisco, California

## Abstract

**Background:**

Access to cardiac surgical services is limited in low- and middle-income countries. The shortage of cardiac surgical care providers is a major contributor to this limited access to lifesaving care. Task shifting and task sharing allow nonspecialists to perform roles typically reserved for specialists such as cardiothoracic surgeons, cardiac anesthesiologists, and other health care workers with specific training in cardiac surgical care. Task sharing has increased access to care without compromising outcomes in related surgical fields, such as general surgery, orthopedic surgery, neurologic surgery, and obstetrics and gynecologic surgery.

**Methods:**

A scoping review was performed according to the Preferred Reporting Items for Systematic reviews and Meta-Analyses extension for Scoping Reviews using the databases PubMed/MEDLINE, Embase, World Health Organization Global Index Medicus, and Web of Science. The search was constructed to identify articles specifically addressing task sharing in cardiac surgical care.

**Results:**

Four relevant articles were identified. Only 1 focused on task sharing specifically by cardiac surgeons; this was a case study of 2 surgeons at a single hospital who used task sharing with junior surgeons to varying degrees. Two studies discussed task sharing as part of a team-based approach to the management of rheumatic heart disease; 1 study discussed cost-effectiveness in the delivery of cardiac surgical care and included task sharing as 1 approach to cost reduction.

**Conclusions:**

There is a paucity of literature describing the applications and outcomes of task sharing in cardiac surgery in variable-resource contexts. Here, we present a scoping review summarizing the literature on experiences with task sharing for cardiac surgical care.

## Graphical abstract

**Figure:**
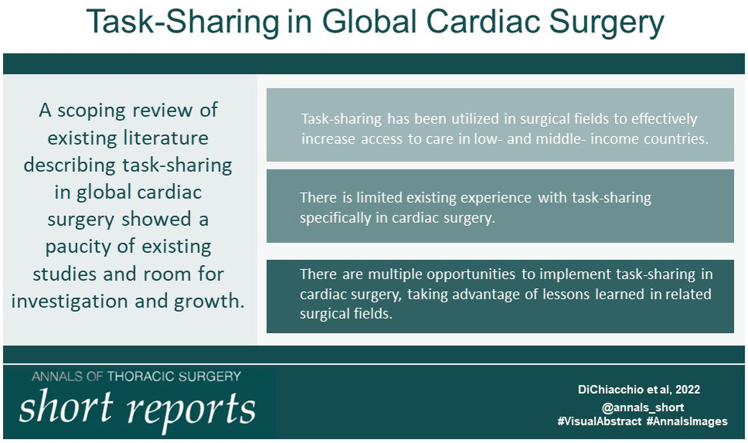

**In Short**

▪ Task sharing has been used to effectively increase access to care in low- and middle-income countries in the fields of obstetrics and gynecologic, general, neurologic, and orthopedic surgery.
▪ Experience with task sharing in cardiac surgery is limited: however, it is an important area for exploration to expand global access to cardiac surgical care.

Task sharing and task shifting are integral to delivery of accessible and effective global health care. Task sharing is the practice in which tasks typically performed by highly specialized personnel are delegated to practitioners with shorter but more focused training with supervision by a specialist. Successful examples of task-sharing programs have been described in various surgical subspecialties.^1^^,^^2^ Whereas task sharing and task shifting are commonly used interchangeably, the key distinguishing feature of task sharing is the continued responsibility and supervision, in some capacity, of the fully trained (physician) member of the team.^1^^,^^3^ Task sharing is not mutually exclusive of team-based care; task sharing is generally adopted when insufficient staffing is available to allow team-based care composed of multiple specialists. Task sharing and team-based care are commonly integrated as capacity is built.^4^

A randomized, prospective trial performed in Sierra Leone compared outcomes in elective inguinal hernia repairs with mesh performed by medical doctors or associate clinicians who have abbreviated medical training between that of nurses and doctors in a particular area of general surgery. This study demonstrated noninferiority of associate clinicians compared with doctors in safety and efficacy with similar 1-year hernia recurrence rates.^5^ Beard and coworkers^6^ compared surgical outcomes in Tanzania during the calendar year 2012 performed by non-physician clinicians (NPCs) compared with physicians. Nonobstetric major operations across 7 district hospitals were included; the most common procedures were groin hernia repair, prostatectomy, exploratory laparotomy, and hydrocelectomy. There was no difference in the postoperative morbidity or mortality between the 2 groups.

Similarly, task sharing has been successfully applied in orthopedic surgery and neurosurgery, primarily in the treatment of emergency infections and traumatic injuries.^7^, ^8^, ^9^, ^10^ Perhaps the most studied surgical field in terms of global task-sharing capacity is obstetrics and gynecologic surgery. Procedures performed include safe abortion services, cesarean deliveries, tubal ligations, and elective male circumcisions. The World Health Organization issued comprehensive guidelines and recommendations surrounding task sharing as a means to increase access for safe abortion services in 2019; their supporting research centers physicians as critical participants in task sharing with midlevel providers such as nurses and midwives to ensure consistent and safe access to abortion care.^11^ In Bangladesh, nonphysician providers including nurses, paramedics, and family welfare visitors have been trained to perform “menstrual regulation services” by manual vacuum aspiration up to 10 weeks since 1979. One study identified barriers to implementation despite government support; these included primarily barriers due to lack of skilled providers or materials, especially lack of equipment for manual vacuum aspiration.^12^ Medical abortions are supervised and require medications prescribed by midwives in many countries, including Sweden and Tunisia.^13^^,^^14^ A scoping review published in 2019 included 15 articles studying performance of NPCs in cesarean births in sub-Saharan Africa; NPCs performed 50% to 94% of cesarean deliveries with outcomes comparable to those of medical doctors.^15^ There are 9 hospitals in Sierra Leone where both associate clinicians and medical doctors perform cesarean deliveries. Results from all 9 hospitals were included in a prospective multicenter noninferiority study focusing on short-term (30-day) outcomes, defined by maternal mortality, maternal morbidity, and perinatal events.^16^ In this cohort of 1161 patients, outcomes after cesarean delivery by associate clinicians were found to be noninferior to those performed by medical doctors, further bolstering support for task sharing to improve access to emergency obstetrical services.

By comparison, the role of task sharing in cardiac surgery has not been well defined. Effective cardiac surgical programs rely on using a broad array of highly trained and specialized practitioners. Cardiac-trained surgeons, surgical assistants, anesthesiologists, cardiologists, nurses, intensivists, perfusionists, technicians, and echocardiographers/sonographers are critical to the delivery of cardiac surgical care. Each role has the potential to use task sharing to safely increase access to care. Here, a scoping review is performed to evaluate the existing literature regarding task sharing in global cardiac surgery.

## Material and Methods

A scoping review was performed according to the Preferred Reporting Items for Systematic reviews and Meta-Analyses extension for Scoping Reviews using the databases PubMed/MEDLINE, Embase, World Health Organization Global Index Medicus, and Web of Science.^17^ A scoping review is a type of literature review that seeks to “scope” the literature by identifying gaps in research and generating research questions. Search terms were selected to include the phrase *task sharing* in combination with multiple integral perioperative services required for cardiac surgical care, including anesthesia, surgery, perfusion, and echocardiography. Combinations of terms and number of results for each term are included in the Supplemental Table. Search results were reviewed manually by 2 independent reviewers and conflicts resolved by a third independent reviewer. Articles written in English, French, and Spanish were included. Review articles, editorials, and commentaries were included. There were no time periods or geographic regions excluded.

## Results

Four relevant articles were identified. One study focused on task sharing specifically by cardiac surgeons.^18^ Two studies discussed task sharing as a component of a team-based approach to the management of rheumatic heart disease.^19^^,^^20^ The final study centered cost-effectiveness in the delivery of cardiac surgical care, including the use of task sharing as 1 approach to cost reduction.^21^ There were no articles identified focusing on task sharing within cardiac surgical anesthesia teams, perfusion, or echocardiography.

The Narayana Health City Cardiac Hospital case study reported on the outcomes of 2 senior cardiac surgeons at a single cardiovascular center in India. The 2 surgeons had similar workloads, patient populations, and experience; however, they varied in the degree to which they used task sharing in their clinical practice.^18^ Task sharing in this study was performed by a junior surgeon working in an apprentice-type position to the senior surgeon. Allowing the junior surgeon to perform less critical portions of coronary artery bypass grafting and valve replacement surgeries, including sternotomies and chest closures as well as internal mammary harvest, freed the time of the senior surgeon to perform more operations in a given day or week, to meet with a higher number of patients for outpatient evaluation for surgery, and to manage inpatients admitted to the ward and intensive care unit. The authors estimated that surgical volume would drop by 25% to 30% without the institution-wide adoption of task sharing among the cardiac surgical team. In retrospectively reviewing outcomes from isolated coronary artery bypass grafting procedures during a 10-month period, this study found no significant difference in patient characteristics or outcomes. The performance of the senior surgeon who used task sharing more readily (70% compared with 43% of time in the operating room) was found to be noninferior to that of the senior surgeon who used task sharing in a more restrictive fashion. In-hospital mortality was 0.74% compared with 1.56% (*P* = .36), and postoperative length of stay was 7.41 days compared with 7.81 days (*P* = .46). The authors highlighted the important fact that task sharing in this study was performed by junior surgeons who had still completed surgical training and were working with senior surgeons to refine their skills. The safety of surgical task sharing depends on the training and skill set of the individuals performing the task-sharing duties, as highlighted in the discussion of this study.

A systematic review published in 2019 studied the role of task sharing in the diagnosis and management of rheumatic heart disease in low- and middle-income countries.^19^ In this systematic review, 212 studies were identified that when screened yielded 20 candidate studies. On detailed review, all studies were excluded because of focusing only on the performance of diagnostic echocardiography by nonphysician workers without further inclusion of task-sharing examples. The authors concluded that there is a lack of evidence for task sharing in capacity building specifically within the scope of rheumatic heart disease. An editorial published that same year discussed the potential of a team-based approach to rheumatic heart disease in low- and middle-income countries, with task sharing being an important component of implementing these teams.^20^

In terms of cost reduction, task sharing has been identified as 1 component of cost reduction in both the United States and India, specifically within cardiac surgery.^21^^,^^22^ Time-driven activity-based costing methods have been used to analyze the impact of such changes. In a case study analyzing cost in India and the United States, a primary cost advantage in the Indian heart hospitals included task sharing with personnel with fewer credentials or less experience with supervision by highly skilled practitioners for tasks with low risk of complications.^21^

## Comment

Four articles were identified in this scoping review discussing task sharing in global cardiac surgery. Although there is a paucity of published experience specifically in capacity building by task sharing in global cardiac surgery, there is much to be learned from experience in other surgical specialties. Educational curricula to implement task sharing, for example, have been described in related fields, including surgical skill acquisition of associate clinicians in Sierra Leone.^23^ Task sharing remains an important tool in implementing robust and effective global cardiac surgical care models. There are several areas to highlight that are amenable to task sharing.

There is limited published experience with task sharing among cardiac surgeons and surgical assistants. This is perhaps the most easily implemented approach to task-sharing cardiac surgical care. Junior cardiac surgeons, general surgeons, and associate clinicians are capable surgical assistants who are able to take over, with training and increasing degrees of independence, portions of operations. Cardiac surgical care teams should take full advantage of these trained individuals’ surgical abilities, particularly in areas with a low surgeon-patient ratio.

There were no identified published studies of task sharing within cardiac anesthesiology; however, this is an important area for further exploration. Non–cardiac trained general anesthesiologists and associate clinicians could similarly take on portions of cases requiring cardiac anesthesia with increasing degrees of independence. Similarly, there were no studies specifically addressing perioperative services, including perfusion and echocardiography (especially intraoperative transesophageal echocardiography). This is a second area that should be explored for further task-sharing applications to build a robust cardiac surgical team.

Whereas cardiac surgery is typically viewed as a higher stakes and more resource- and training-intensive specialty, other fields, such as neurosurgery and obstetrics, are no less specialized or critical and have demonstrated effective delivery of care through the application of thoughtful and deliberate task sharing. The Narayana Health City Cardiac Hospital case study highlights the feasibility of task sharing specifically within the cardiac surgical operating room, with no compromise in patient outcomes. This should be taken as an encouraging experience that demonstrates the safety of task sharing in building cardiac surgical capacity and enabling access to cardiac surgical care for all who need it. Financial and ethical considerations in the implementation of task sharing are several. Overall, task sharing should lead to a reduction in the cost of training and retaining team members. Ethical issues are complex, and it is important to ensure that task sharing does not result in the delivery of poor-quality care. This requires structured training, oversight, and accreditation as poor-quality care is worse than no care, even as care by task-shifted or task-sharing individuals is better than no care.^24^

### Limitations

There are several limitations to this review. This is a scoping review, which is designed to identify gaps in existing literature and to generate research questions. Accordingly, in this case, only a small number of articles were identified, leaving limited evidence from which to draw conclusions. In addition, articles may have been missed, for example, on the basis of language. Any articles that describe task sharing by similar terms without explicitly using the term *task sharing* would have been missed in this search.

### Conclusion

There is currently limited published experience with task sharing in global cardiac surgery. Access to cardiac surgical services, however, is limited around the world in large part owing to a shortage of cardiac surgical care providers. This scoping review aims to provide a groundwork for exploring task sharing specifically within cardiac surgery.

## References

[1] Meara J.G., Leather A.J., Hagander L. (2015). Global Surgery 2030: evidence and solutions for achieving health, welfare, and economic development. Lancet.

[2] Hoyler M., Hagander L., Gillies R. (2015). Surgical care by non-surgeons in low-income and middle-income countries: a systematic review. Lancet.

[3] Smith M. (2018). Is task sharing preferred to task shifting in the provision of safe surgical care?. Surgery.

[4] Lester L., Njuguna B., Vedanthan R., Kpodonu J. (2022). Global Cardiac Surgery Capacity Development in Low and Middle Income Countries.

[5] Ashley T., Ashley H., Wladis A. (2021). Outcomes after elective inguinal hernia repair performed by associate clinicians vs medical doctors in Sierra Leone: a randomized clinical trial. JAMA Netw Open.

[6] Beard J.H., Oresanya L.B., Akoko L., Mwanga A., Mkony C.A., Dicker R.A. (2014). Surgical task-shifting in a low-resource setting: outcomes after major surgery performed by nonphysician clinicians in Tanzania. World J Surg.

[7] Mkandawire N., Ngulube C., Lavy C. (2008). Orthopaedic clinical officer program in Malawi: a model for providing orthopaedic care. Clin Orthop Relat Res.

[8] Flores M., Kisitu D., Peck C., Amick M., Yu K., Socci A. (2021). Orthopaedic surgical task-shifting and its impact on time-to-surgery in western Uganda. Eur J Clin Biomed Sci.

[9] Robertson F.C., Esene I.N., Kolias A.G., Collaborative Working Group (2019). Task-shifting and task-sharing in neurosurgery: an international survey of current practices in low- and middle-income countries. World Neurosurg X.

[10] Robertson F.C., Briones R., Mekary R.A. (2019). Task-sharing for emergency neurosurgery: a retrospective cohort study in the Philippines. World Neurosurg X.

[11] Kim C., Sorhaindo A., Ganatra B. (2020). WHO guidelines and the role of the physician in task sharing in safe abortion care. Best Pract Res Clin Obstet Gynaecol.

[12] Sultana N. (2020). Task-sharing in menstrual regulation services: implementation efforts and lessons learned in Bangladesh. Int J Gynaecol Obstet.

[13] Endler M., Cleeve A., Sääv I., Gemzell-Danielsson K. (2020). How task-sharing in abortion care became the norm in Sweden: a case study of historic and current determinants and events. Int J Gynaecol Obstet.

[14] Hajri S., Belhadj H. (2020). The role of midwives in first-trimester abortion care: a 40-year experience in Tunisia. Int J Gynaecol Obstet.

[15] Matinhure S., Chimbari M.J. (2019). Barriers and enablers to task shifting for caesarean sections in sub-Saharan Africa: a scoping review. Afr J Reprod Health.

[16] van Duinen A.J., Kamara M.M., Hagander L. (2019). Caesarean section performed by medical doctors and associate clinicians in Sierra Leone. Br J Surg.

[17] Tricco A.C., Lillie E., Zarin W. (2018). PRISMA Extension for Scoping Reviews (PRISMA-ScR): checklist and explanation. Ann Intern Med.

[18] Gupta B., Huckman R.S., Khanna T. (2015). Task shifting in surgery: lessons from an Indian heart hospital. Healthc (Amst).

[19] Abdullahi L.H., Smit I., Engel M.E., Watkins D.A., Zühlke L.J. (2019). Task sharing in the diagnosis, prevention, and management of rheumatic heart disease: a systematic review. Glob Heart.

[20] Lee S.S., Vedanthan R. (2019). Beyond sharing and shifting: raising the bar for global rheumatic heart disease control. Glob Heart.

[21] Erhun F., Kaplan R.S., Narayanan V.G. (2020). Are cost advantages from a modern Indian hospital transferable to the United States?. Am Heart J.

[22] Vervoort D., Swain J.D. (2022). Cost reduction in cardiothoracic surgery: the case for time-driven activity-based costing. Ann Thorac Surg.

[23] Liu B., Hunt L.M., Lonsdale R.J. (2018). Comparison of surgical skill acquisition by UK surgical trainees and Sierra Leonean associate clinicians in a task-sharing programme. BJS Open.

[24] Kruk ME, Gage AD, Arsenault C, et al. High-quality health systems in the Sustainable Development Goals era: time for a revolution. *Lancet Glob Health*. 2018;6:e1196-e1252. Published correction appears in *Lancet Glob Health*. 2018;6:e1162. https://doi.org/10.1016/S2214-109X(18)30386-3 10.1016/S2214-109X(18)30386-3 PMC7734391 30196093

