# Supplementary material for: Task Sharing in Global Cardiac Surgery: A Scoping Review
Source: Ann Thorac Surg Short Rep. 2022 Dec 12;1(1):216–20. doi: 10.1016/j.atssr.2022.12.004 (PMC11708438; doi:10.1016/j.atssr.2022.12.004)
Supplement: Supplemental Table [file mmc1.docx]

**Supplemental Materials**

**Supplemental Table 1**. Scoping review literature search strategy.

| **Search String** | **Results** |
| --- | --- |
| ***PubMed/MEDLINE*** | |
| ("task-sharing"[tiab] OR "task sharing"[tiab] OR "task-shifting"[tiab] OR "task shifting"[tiab] OR "non-clinician*"[tiab]) AND ("heart surg*"[tiab] OR "cardiothoracic surg*"[tiab] OR "cardiovascular surg*"[tiab] OR "cardiac surg*"[tiab] OR "cardiology"[tiab] OR "echocardiograph*"[tiab] OR "perfusion"[tiab] OR "cardiovascular anesthesia"[tiab] OR "cardiothoracic anesthesia"[tiab] OR "cardiac anesthesia"[tiab]) | 22 |
| ***EMBASE*** | |
| ('task-sharing':ti,ab OR 'task sharing':ti,ab OR 'task-shifting':ti,ab OR 'task shifting':ti,ab OR 'non-clinician*':ti,ab) AND ('heart surg*':ti,ab OR 'cardiothoracic surg*':ti,ab OR 'cardiovascular surg*':ti,ab OR 'cardiac surg*':ti,ab OR 'cardiology':ti,ab OR 'echocardiograph*':ti,ab OR 'perfusion':ti,ab OR 'cardiovascular anesthesia':ti,ab OR 'cardiothoracic anesthesia':ti,ab OR 'cardiac anesthesia':ti,ab) | 44 |
| ***World Health Organization Global Index Medicus*** | |
| ("task-sharing" OR "task sharing" OR "task-shifting" OR "task shifting" OR "non-clinician*") AND ("heart surg*" OR "cardiothoracic surg*" OR "cardiovascular surg*" OR "cardiac surg*" OR "cardiology" OR "echocardiograph*" OR "perfusion" OR "cardiovascular anesthesia" OR "cardiothoracic anesthesia" OR "cardiac anesthesia") | 0 |
| ***Web of Science*** | |
| (TI=("task-sharing" OR "task sharing" OR "task-shifting" OR "task shifting" OR "non-clinician*") OR AB=("task-sharing" OR "task sharing" OR "task-shifting" OR "task shifting" OR "non-clinician*")) AND (TI=("heart surg*" OR "cardiothoracic surg*" OR "cardiovascular surg*" OR "cardiac surg*" OR "cardiology" OR "echocardiograph*" OR "perfusion" OR "cardiovascular anesthesia" OR "cardiothoracic anesthesia" OR "cardiac anesthesia") OR AB=("heart surg*" OR "cardiothoracic surg*" OR "cardiovascular surg*" OR "cardiac surg*" OR "cardiology" OR "echocardiograph*" OR "perfusion" OR "cardiovascular anesthesia" OR "cardiothoracic anesthesia" OR "cardiac anesthesia")) | 15 |
